# Genome-wide identification of MAPK family genes and their response to abiotic stresses in tea plant (*Camellia sinensis*)

**DOI:** 10.1515/biol-2022-0466

**Published:** 2022-09-08

**Authors:** Xinhao Liu, Min Zhao, Caihua Gu, Haodong Jiang, Junyan Sun, Jie Li

**Affiliations:** Central Laboratory, Xinyang Agriculture and Forestry University, Xinyang, Henan, 464001, China; College of Agronomy, Xinyang Agriculture and Forestry University, Xinyang, Henan, 464001, China

**Keywords:** tea plant, mitogen-activated protein kinase, gene expression, abiotic stresses

## Abstract

Mitogen-activated protein kinase (MAPK) cascades are conserved and universal signal transduction modules that play important roles in regulating stress responses in plants. Although MAP3K, MP2K, and MPK family in tea plant (*Camellia sinensis*) have been investigated, little is known about *MPK* family genes responding to various abiotic stresses in tea plant. In this study, we performed a comprehensive genome-wide analysis of the tea plant *MAPKs* (*CsMPKs*) family gene based on the genomic data of tea plants by bioinformatics-based methods. Here, 21 putative *CsMPK* genes were identified in the tea plant and divided into 4 subfamilies according to the homologous to *Arabidopsis* and their phylogenetic relationships. The gene structure and conserved motifs of these *CsMPKs* in the same group showed high similarity, suggesting that they were highly conserved and might have a similar function. The expression profiles of the *CsMPK* genes were further investigated by quantitative real-time reverse transcription PCR, indicating that many *CsMPK* genes were involved in response to cold, drought, heat, or heat combined with drought treatment, suggesting their potential roles in abiotic stress responses in tea plant. These results would provide valuable information for further exploring the functional characterization of *CsMPK* genes in tea plants.

## 1 Introduction

As sessile organisms, plants often encounter various adverse conditions, such as high and low temperatures, drought, salt stresses, and pathogen invasion, during their growth and development. To coordinate the biotic and abiotic stresses, plants have evolved complex signaling networks to perceive and transmit environmental stimuli. Mitogen-activated protein kinase (MAPK) cascades play vital roles in controlling intracellular response to extracellular signals [1].

MAPK cascades classically are composed of three subsequently protein kinases, namely MAP kinase kinase kinase (MAPKKK/MAP3K/MEKK), MAP kinase kinase (MAPKK/MKK/MEK), and MAP kinase (MAPK/MPK), but sometimes contain an upstream MAP4K [2]. In general, MAPKs are phosphorylated by MAPK kinase at their conserved threonine and tyrosine residues in the activation loop (T-loop), and in turn, MAPKK is phosphorylated in the S/T-X_3–5_-S/T motif of their activation loop by MAPKKKs when development or environmental signals incurred [3]. Compared with MAPKKKs and MAPKKs, MAPKs act at the bottom of MAPK cascades showing more complexity and sequence diversity. Plant MAPKs can be divided into four groups, including A, B, C, and D subfamilies based on the phosphorylation motif and phylogenetic relationships of amino acid sequence. Members of the A, B, and C subfamilies have a TEY motif in their active sites, while members in the D subfamily possess a TDY motif in their phosphorylation site [1].

In plants, numerous researches demonstrated that MPKs play indispensable regulatory roles in response to abiotic stresses [4]. Such as MPK3 and MPK6 were reported to play a critical role in response to drought, cold, salt, and heat stress in *Arabidopsis* [4,5], Jammes et al. demonstrated that AtMPK9 and AtMPK12 were preferentially and highly expressed in guard cells and positively regulated ROS-mediated ABA signaling [6]. A role for the MAPK module consisting of MEKK1-MKK1/2-MPK4 has been confirmed in response to drought, cold, and salt stresses in *Arabidopsis* [7,8]. In rice, OsMPK5 was shown to be a double-faced player in the stress response network: a positive regulator of abiotic stress tolerance and ABA-mediated defense responses to brown spots but a negative regulator of ET-controlled resistance to *Magnaporthe oryzae* [9]. *OsMPK33* was demonstrated to play a negative role in salt tolerance through unfavorable ion homeostasis [10]. The MKK1–MPK4 module was reported to mediate salt signaling in rice [11]. OsMKK6 and OsMPK3 constitute a moderately low-temperature signaling pathway and regulate the tolerance to cold stress in rice [12]. In addition, in maize, MPK4, MPK5, MPK6, and MPK7 were active by diverse stresses, such as drought, low temperature, and salt [13].

In light of the importance of MAPK genes, the identification and characterization of MAPK members have been conducted in various plant species. Such as *Arabidopsis thaliana* genome contains 20 MAPKs [3], there are 17 MAPKs in rice (*Oryza sativa*) genome [14], 54 MAPK genes were identified in wheat (*Triticum aestivum*) [15], 28 MAPKs in cotton (*Gossypium raimondii*) [16], and 28 MAPKs in sunflower (*Helianthus annuus*) [17]. Tea plant (*Camellia sinensis*), an evergreen woody plant, is an important economic crop that is widely distributed in subtropical to tropical climate regions [18]. Due to the local climate changes, tea plant frequently experiences various environmental stresses during its lifecycle, and heat, drought, and low-temperature stresses are the main factors that affect the yield and quality of tea products [19,20]. Recently, high-quality genome sequencing data for tea plants were presented, which provides convenience for further understanding the tea plant *CsMPK* gene family [21,22,23]. In the present study, 21 *CsMPK* genes were identified using the tea plant genome by exploring the genomic data. The analysis of identified *CsMPK* genes in the sequence features, phylogenetic relationships, *cis*-elements in promoters, and dynamic expression patterns in response to various abiotic stresses was conducted. The results of this study provided valuable information for further investigation of tea plant *MPK* gene family.

## 2 Materials and methods

### 2.1 Identification and characterization of *CsMPK* gene family

The identification of the *CsMPK* gene family of tea plant was conducted according to the method described by Wang et al. with some modifications [24]. The MPK protein sequences of *A. thaliana*, *O. sativa*, *Vitis vinifera*, and *Populus trichocarpa* were downloaded from TAIR (http://www.Arabidopsis.org), the Rice Genome Annotation Project (http://rice.plantbiology.msu.edu/index.shtml), the *V. vinifera* proteome 12 × database (http://www.genoscope.cns.fr/externe/GenomeBrowser/Vitis/), and Ensembl database (http://plants.ensembl.org), respectively. Whole genome sequences of tea plant were downloaded from the Tea Plant Genome Database (http://tpia.teaplant.org/index.html). The MPK sequences of *A. thaliana*, *O. sativa*, *V. vinifera*, and *P. trichocarpa* were used as queries to search against tea plant proteins using BLASTP program with an *e*-value of 1 × 10^−5^ as the threshold. All the acquired MPK protein sequences from the four plants mentioned above were used to build the local Hidden Markov Model-based searches (HMMER) that were used to identify the CsMPKs in tea plant with the threshold of *E* < 1 × 10^−20^. Then, the BLASTP results were integrated with the HMMER hits and parsed by manual editing to remove redundant. Furthermore, these identified CsMPK members were confirmed by CDD (https://www.ncbi.nlm.nih.gov/cdd/), PFAM (http://pfam.xfam.org/), InterProScan database (http://www.ebi.ac.uk/interpro/), and SMARAT (http://smart.embl-heidelberg.de/). The molecular weight (MW) and isoelectric point (pI) of the CsMPKs were predicted using ProtParam (http://web.expasy.org/protparam/).

### 2.2 Sequence alignments and phylogenetic tree construction of CsMPK genes

Multiple alignments of amino acid sequences of MPKs were performed using ClustalW program with the default parameters [25]. Phylogenetic tree of MPKs from *C. sinensis*, *A. thaliana*, *O. sativa*, *V. vinifera*, and *P. trichocarpa* was generated by MEGA 6.0 software using a neighbor-joining (NJ) method with 1,000 bootstrap replicates [26], which was visualized in Figtree (http://tree.bio.ed.ac.uk/software/figtree/).

### 2.3 Gene structure and conserved motif analysis

The exon/intron structure of the *CsMPK* genes was retrieved from a gene annotation file (http://www.plantkingdomgdb.com/tea_tree/data/gff3/), and the diagrams were displayed by comparing CDSs and their corresponding gene sequences from genome using the online program GSDS 2.0 (http://gsds.cbi.pku.edu.cn/) [27]. The conserved motifs of CsMAPKs were analyzed by the program of MEME with the following parameters: any number of repetitions, maximum of 10 motifs, and an optimum motif width of 6–50 amino acid residues [28].

### 2.4 *cis*-element analysis of putative promoter regions

The upstream sequences (1.5 kb) of *CsMPK* genes were submitted to the PlantCARE database (http://bioinformatics.psb.ugent.be/webtools/plantcare/html/) to predict various *cis*-regulatory elements according to consensus sequences, positional matrices, and individual sites on particular promoter sequences [29].

### 2.5 Plant materials and treatments

Two-year-old cutting seedlings of the tea cultivar “Longjing43” were grown in a controlled environment chamber maintained for 12 h day (25 ± 3°C) and 12 h night (20 ± 3°C), and 75% relative humidity for 2 weeks. Then, cold, heat, drought, and heat combined with drought stress treatments were performed. Untreated tea plants were used as control. For cold treatment, the tea plants were kept in a growth chamber at 4°C. The first and second tender leaves were harvested 24 and 48 h after the treatment from control and cold-treated plants. The treatments of heat, drought, and heat combined with drought stress were conducted simultaneously. For heat treatment, the tea plants were transferred to chambers maintained at 38°C. For drought treatment, the tea plants were irrigated with 20% (w/v) polyethylene glycol (PEG) 6000. For heat combined with drought stress, the tea plants were irrigated PEG 6000 and then quickly put in chambers at 38°C. The first and second tender leaves treated with drought, heat, heat combined with drought stresses, and control leaves were collected 24 h post-treatment, respectively. The collected leaves were immediately frozen in liquid nitrogen and then stored at −80°C for gene expression analysis. For each treatment, three independent biological replicates were performed.

### 2.6 Total RNA isolation and quantitative real-time reverse transcription PCR (qRT-PCR) expression analysis

Total RNA was extracted from young leaves of tea plants using the “TaKaRa MiniBEST Plant RNA Extraction” Kit (TaKaRa, Dalian, China) in accordance with the manufacturer’s protocol. RNase-free DNaseI was added to each sample to digest the genomic DNA. Then, cDNA was synthesized following the kit instruction (Promega Corp., Madison, WI, USA) and stored at −20°C. The primer sequences used in this experiment were designed based on gene sequences and the Beacon designer software (NJ, USA). CsGAPDH was used as the reference gene [30]. The primer sequences are shown in Table S1. The qRT-PCR was performed on a CFX96 Real-Time PCR Detection System (Bio-Rad Laboratories, Inc.) with three independent biological replicates. The reaction conditions were: denaturation at 95°C for 30 s, 40 cycles of 95°C for 5 s, and 60°C extensions for 30 s. Relative gene expression levels were calculated using the 2^−∆∆CT^ method [31].

## 3 Results

### 3.1 Identification and characterization of *CsMPK* family genes in tea plant

To identify MPK encoding genes from tea plant genome, both BLASTP and Hidden Markov Model (HMM) searches were conducted using *A. thaliana*, *O. sativa*, *V. vinifera*, and *P. trichocarpa* MPK proteins as query sequences and then used these search results to identify all the CsMPK family members by CDD, PFAM, and SMART programs. Finally, a total of 21 CsMPK members were identified from tea plant genome. To distinguish from these identified CsMPKs, we provisionally named them according to the homologous gene in *A. thaliana* (Table 1). The sequence data of all the above CsMPKs are shown in Table S2. The information analysis of CsMPKs showed that the open reading frame (ORF) length for *CsMPK* genes ranged from 850 to 6,765 bp, and they encoded proteins ranging from 284 to 1,482 amino acids in size. The deduced protein MWs ranged from 32.65 to 169.32 kDa, and the protein isoelectric points (pIs) were between 4.9 and 9.37 (Table 1).

**Table 1:** The information of MAPK gene family in tea plant

Gene	Homologous gene	Sequence ID	Group	ORF length (bp)	Protein length (aa)	MW (kDa)	pI
CsMPK1-1	AtMPK1	TEA031435	C	1,262	421	47.94	5.97
CsMPK1-2	AtMPK1	TEA016315	C	1,152	384	44.23	6.70
CsMPK3-1	AtMPK3	TEA026040	A	1,113	372	42.75	5.25
CsMPK3-2	AtMPK3	TEA020852	A	1,092	365	41.68	5.89
CsMPK3-3	AtMPK3	TEA020851	A	850	284	32.65	5.62
CsMPK3-4	AtMPK3	TEA017807	A	2,461	822	91.59	8.64
CsMPK3-5	AtMPK3	TEA017811	A	1,110	371	41.43	6.27
CsMPK4-1	AtMPK4	TEA006273	B	6,765	1,482	169.32	4.90
CsMPK4-2	AtMPK4	TEA021759	B	988	330	38.21	5.41
CsMPK4-3	AtMPK4	TEA006436	B	1,098	367	42.24	6.28
CsMPK4-4	AtMPK4	TEA007103	B	2,379	798	90.21	6.18
CsMPK6	AtMPK6	TEA024415	A	1,516	507	57.24	6.06
CsMPK7	AtMPK7	TEA032724	C	1,105	368	42.22	7.60
CsMPK9-1	AtMPK9	TEA022268	D	1,554	517	59.34	6.75
CsMPK9-2	AtMPK9	TEA015676	D	1,698	569	64.24	6.43
CsMPK15	AtMPK15	TEA012905	D	1,753	587	66.87	6.99
CsMPK16-1	AtMPK16	TEA026883	D	2,916	975	110.3	9.22
CsMPK16-2	AtMPK16	TEA031053	D	1,752	587	66.62	9.09
CsMPK19-1	AtMPK19	TEA018880	D	1,791	599	67.28	9.22
CsMPK19-2	AtMPK19	TEA022253	D	2,363	792	90.12	9.25
CsMPK20	AtMPK20	TEA004137	D	855	286	33.27	9.37

### 3.2 Phylogenetic relationship analysis of *CsMPK* genes

**Figure 1:**
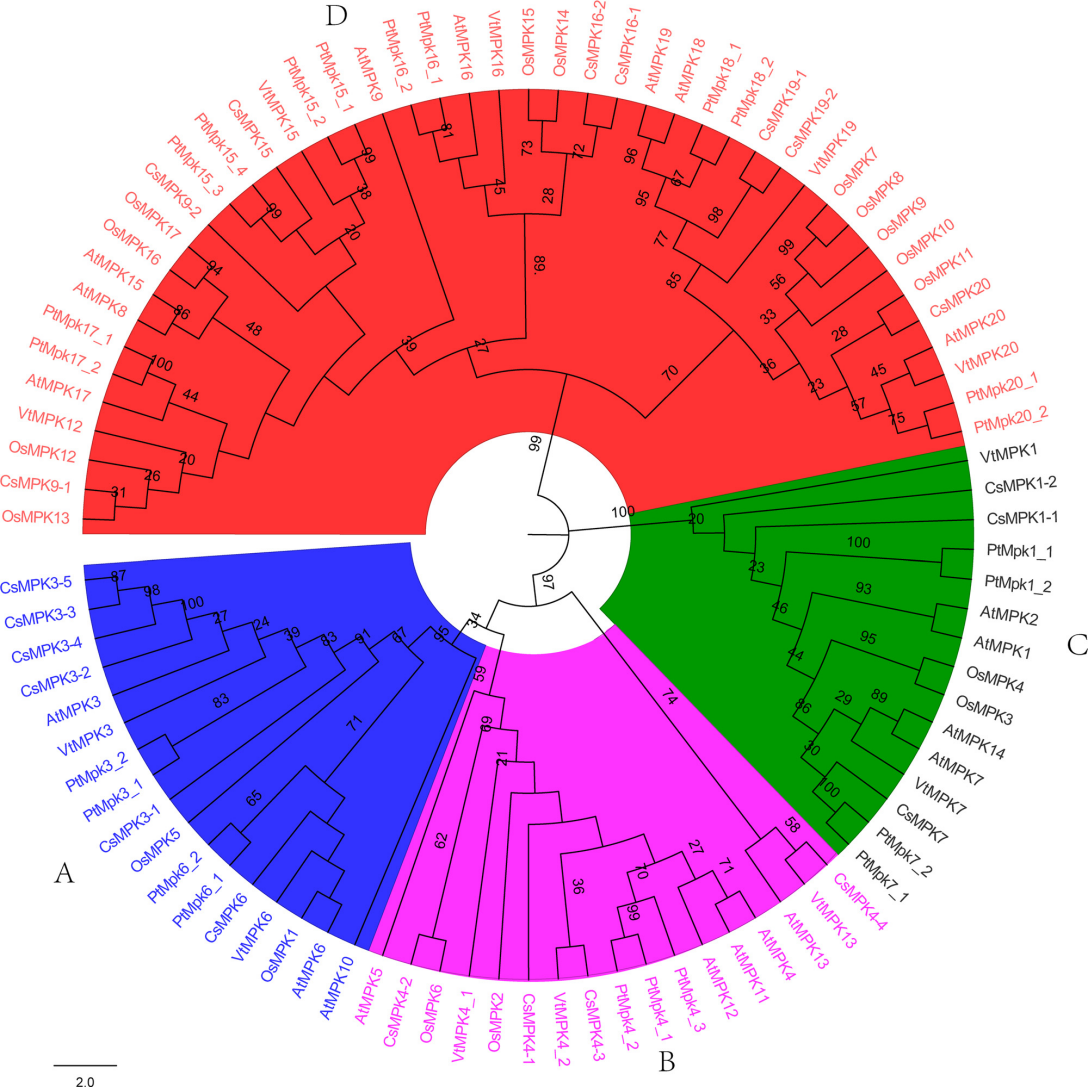
Phylogenetic relationship of putative MPK genes from *A. thaliana* (AtMPK), *O. sativa* (OsMPK), *V. vinifera* (VvMPK), *P. trichocarpa* (PtMPK), and *C. sinensis* (CsMPK). The phylogenetic tree with 100 bootstrap replicates was constructed by MEGA6.0 program with the NJ method. Letters A–D named as different groups of MPKs.

To analyze the phylogenetic relationships of the MPKs, an NJ phylogenetic tree was constructed with the amino acid sequences of 20 AtMPKs from *Arabidopsis*, 17 OsMPKs from *O. sativa*, 12 VvMPKs from grapevine, 23 PtMPKs from poplar, and 21 putative CsMPKs from tea plant (Figure 1). Through phylogenetic analyses, these MPKs were classified into four groups, designated as A, B, C, and D. Group D was the first largest clade, including eight CsMPKs, followed by group A (six members). Group C (three members) had the fewest number of CsMPKs; the rest belonged to group B, containing four CsMPKs. CsMPKs belonging to groups A, B, and C possess a TEY motif, and the D group has a TDY motif at the activation site (Figure S1).

### 3.3 Conserved motifs analysis of CsMPKs

**Figure 2:**
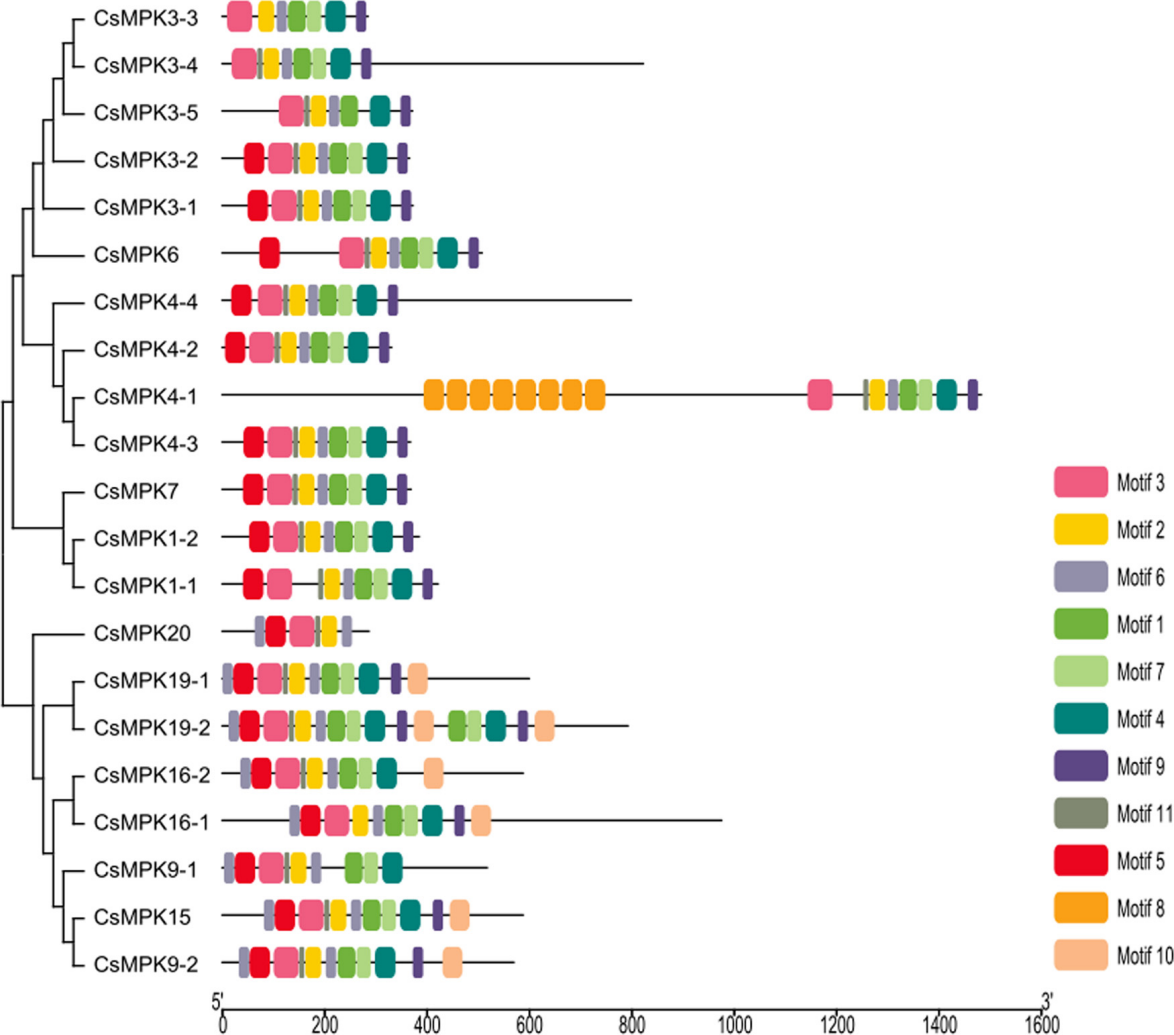
The distribution of conserved motifs of CsMPKs from tea plant according to the phylogenetic relationship. The motifs were identified by MEME program with the complete amino acid sequences of the 21 CsMPKs. Each motif is represented by one color box.

The conserved protein domains in CsMPKs were identified and compared by the online MEME program. A total of 11 conserved motifs were detected (Figure 2). The lengths of the identified motifs varied from 11 to 50 amino acids (motif logos shown in Figure S2). Two CsMPKs (CsMPK4-1 and CsMPK19-2) contained the maximum number of motifs (16), while the minimum number of motifs (6) occurred in CsMPK20. Furthermore, all of CsMPKs contained motifs 2, 3, and 6 (contained the TXY signature motif), but motif 8 was found only in CsMPK4-1. The alignment analysis and sequence logo revealed that the sequences of CsMPKs in the motif regions were highly conserved (Figures S1 and S2). In addition, the members in the same subfamily were found to share similar conserved motifs. For example, the majority of CsMPKs in groups A, B, and C possessed 9 motifs, whereas most members in group D had at least 10 motifs. Interestingly, motif 6 in group D members occurred two times. These CsMPKs in the same subgroup showed similar motif distribution indicating that these genes might have relatively high conservation.

### 3.4 Gene structure analysis of *CsMPK* genes

**Figure 3:**
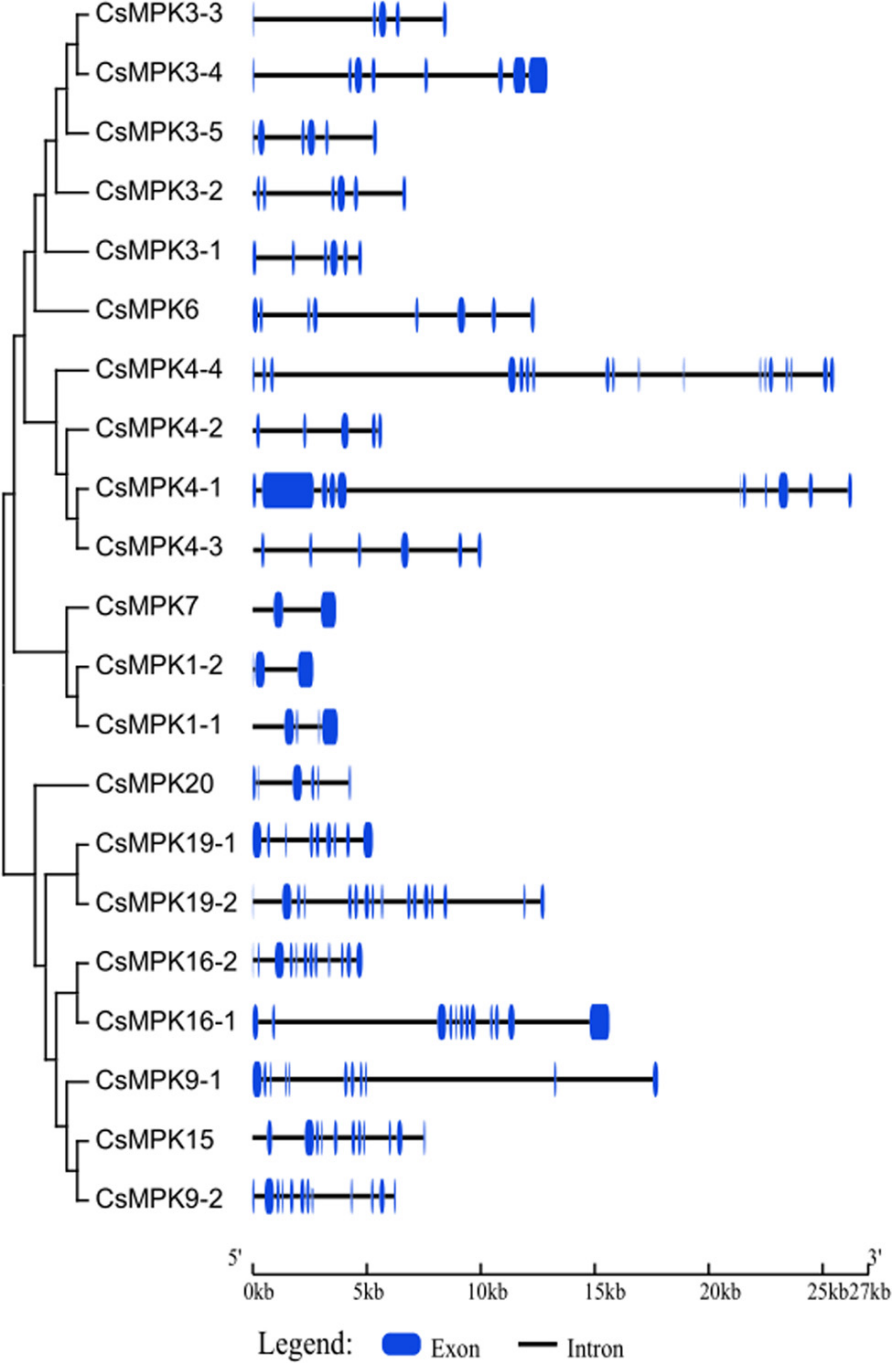
Phylogenetic relationship and gene structure of *CsMPK* genes. The phylogenetic tree was created using MEGA6.0 program with the NJ method. Exons and introns are indicated by blue bars and thin lines, respectively. Gene models were drawn to scale as indicated on bottom.

Gene structures of the identified 21 *CsMPK* genes were analyzed by aligning tea plant genomic DNA sequences. As shown in Figure 3, the exon/intron structures of *CsMPK* genes could be divided into four groups on the basis of their phylogenetic relationship, and those *CsMPK* genes belonged to the same clade and shared similar intron/exon organization structures. In group A, the number of introns in *CsMPKs* varied from 4 to 7. Intron numbers in group B varied widely, from 4 (*CsMPK4-2*) to 17 (*CsMPK4-4*). Group C had no more than 3 introns, while group D had at least 10 introns except CsMPK20 and CsMPK19-1, containing 5 and 8 introns, respectively.

### 3.5 Stress-related *cis*-elements analysis of *CsMPK* genes

**Figure 4:**
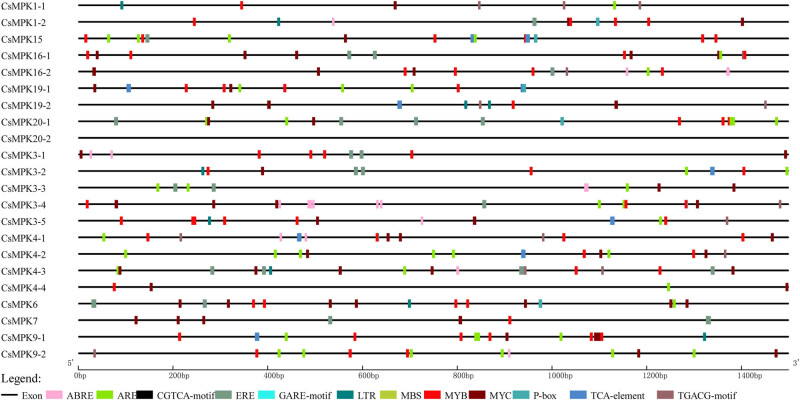
Predicted *cis*-elements in the 1.5 kb upstream promoter regions of *CsMPK* genes. Different *cis*-elements are represented by different colors.

To further explore the regulatory mechanisms and potential functions of *CsMPK* genes under stress conditions, the 1.5 kb promoter sequences of the *CsMPK* genes were used to analyze the *cis*-regulatory elements. Two categories of *cis*-elements were found in the promoter region of *CsMPK* genes (Figure 4 and Table S3). The first class was constituted by hormone-responsive elements, such as ABRE, ERE, and P-box. TGACG-motif and CGTCA-motif are involved in methyl jasmonate responsiveness, which existed in 10 *CsMPKs*. ERE is related to an ethylene-responsive element found in 12 *CsMPKs*. The ABRE element existed in nine *CsMPKs*. The results suggested that some phytohormones (e.g., ABA and JA) are involved in regulating the expression of some *CsMPK* genes. The other category was constituted by abiotic stress response-related *cis*-elements, including LTR, MBS, MYB, MYC, and ARE. MYC responds to abiotic stress signals, which existed in all of *CsMPK* genes. MYB responds to dehydration and ABA signals and were also found in all *CsMPK* genes except *CsMPK3-3*. Eight *CsMPKs* possessed LTRE, which responds to cold, drought, and ABA signals. MBS is involved in drought response and detected in 8 *CsMPKs*.

### 3.6 Expression patterns of *CsMPK* genes under abiotic stresses

**Figure 5:**
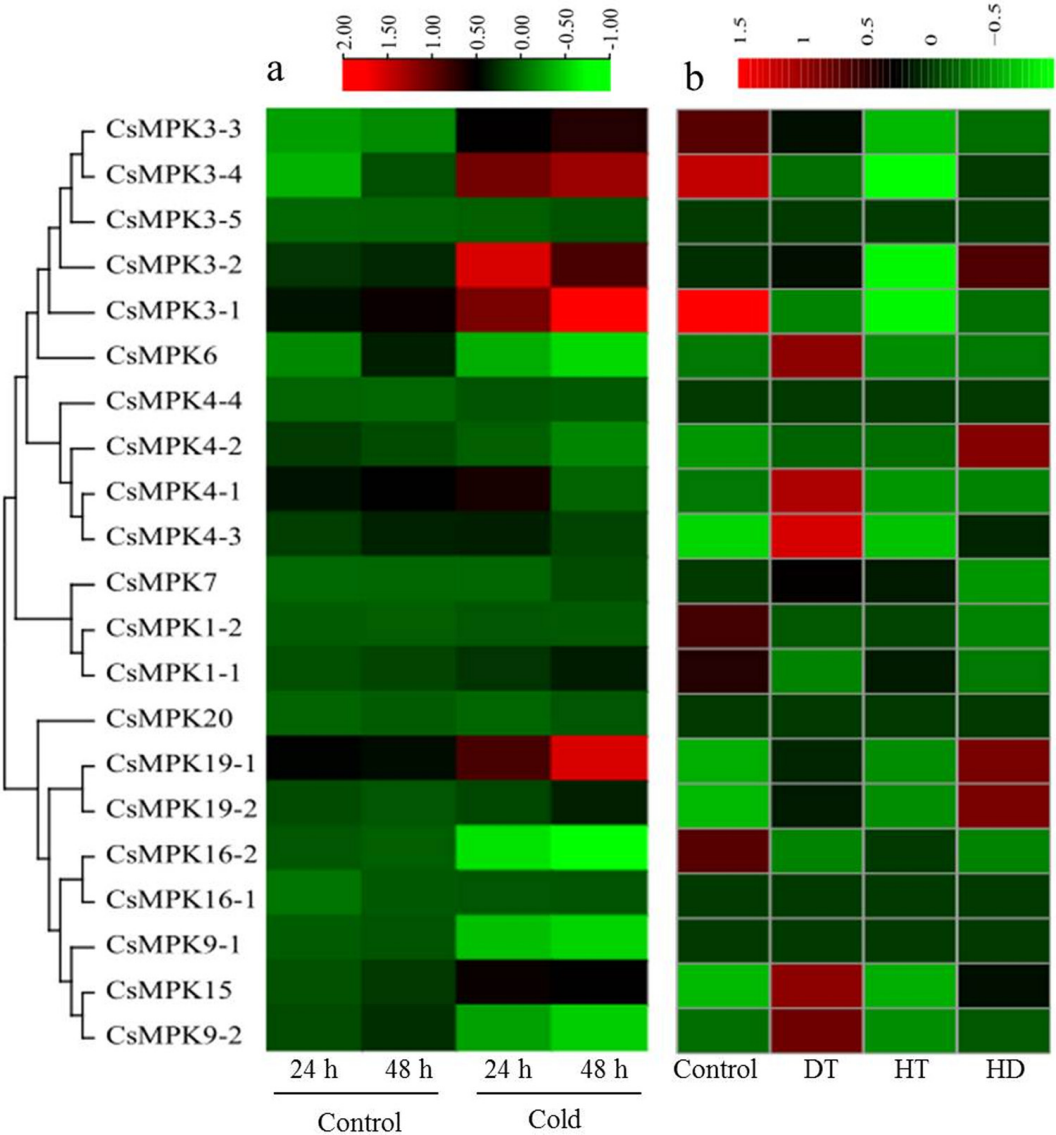
Hierarchical clustering of the expression patterns of *CsMPK* genes in tea plant leaves under cold, drought, heat, and heat combined with drought stresses. The red and green colors indicate higher or lower expression levels, respectively. (a) Heatmap of the *CsMPK* genes expression under cold treatment. (b) Heatmap of the *CsMPK* genes expression under drought, heat, and heat combined with drought treatments. DT, HT, and HD were denoted as drought, heat, and heat combined with drought treatments, respectively.

To examine the potential role of *CsMPK* genes in various abiotic stresses, the expression patterns of all of these genes were investigated under heat, drought, or cold stress conditions (Figure 5a and b). For cold treatment (Figure 5a), the expressions of six *CsMPK* genes (*CsMPK3-1*, *CsMPK3-2*, *CsMPK3-3*, *CsMPK3-4*, *CsMPK4-3*, *CsMPK15*, and *CsMPK19-1*) were induced to present a significant upregulation, while *CsMPK9-1*, *CsMPK9-2*, and *CsMPK16-2* were downregulated under cold stress condition. *CsMPK4-1* was upregulated at 24 h of treatment and then downregulated at 48 h. Among the upregulated *CsMPK* genes, four genes belonged to group A, one in group B, and another two in group D. In response to drought treatment (Figure 5b), most group A and group C members were downregulated, whereas *CsMPK* genes in groups B and D showed a significantly upregulated expression. For heat treatment, only six *CsMPK* genes (*CsMPK3-3*, *CsMPK3-4*, *CsMPK3-2*, *CsMPK3-1*, *CsMPK1-2*, and *CsMPK16-2*) displayed downregulation, and no gene was found to show significant up-regulation (Figure 5). Under heat combined with drought treatments (Figure 5b), six *CsMPK* genes (*CsMPK3-2*, *CsMPK4-2*, *CsMPK4-3*, *CsMPK19-1*, *CsMPK19-2*, and *CsMPK15*) were significantly upregulated, and another six genes (*CsMPK3-3*, *CsMPK3-4*, *CsMPK3-1*, *CsMPK1-1*, *CsMPK1-2*, and *CsMPK16-2*) displayed a downregulated expression trend (Figure 5b). In addition, no changes in the expression of three *CsMPK* genes (*CsMPK3-5*, *CsMPK4-4*, and *CsMPK20*) were observed under cold, heat, drought, and heat combined with drought treatments. Notably, *CsMPK3-2* showed differential expression under three stress conditions (cold, heat, and heat combined drought), indicating that it might play an important role in response to several stresses.

## 4 Discussion

Due to the importance of plant MPKs in multifaceted biological processes such as growth, development, and stress responses [32,33], lots of MPK family members were identified in many plants through whole genome sequencing [34,35]. Recently, the tea plant genome database has been published [21,22,23], which provides the basis for the comprehensive analysis of MAPK cascade members. As the upstream components of the MAPK cascade, MAPKKK family members have been described in detail [36]. Chatterjee et al. analyzed the expression profiles and regulatory network of the MAPK cascade (MAPKK–MAPK) gene family of the tea plant [37]. Tea is the main industrial crop of the Xinyang district, which is often challenged by cold, heat, drought, and heat combined with drought stresses. To implore the tolerance mechanism of tea plant to above-mentioned stresses, we performed a detailed analysis of the *CsMPK* family genes and the expression characteristics of these genes under abiotic stresses. In this study, 21 *CsMPK* genes were identified in the tea plant genome. The number of *CsMPKs* is close to the *MPK* gene number in *Arabidopsis* (20 *MPKs*) and poplar (23 *MPKs*) (Nicole et al. identified 21 *PtMPK* genes from *P. trichocarpa* genome [38]. The variation in *PtMPKs* amount within the same species in the two studies might come as a result of different parameters employed during HMM profiling and motif analysis), but the genome size of tea plant (∼2.85 Gb) is approximately 23 times that of the *Arabidopsis* genome (∼125 Mb) and 6 times than poplar genome (∼485 Mb) [39]. The number of *CsMPK* members in tea plant was more than in grapevine (12 members), coffee (12 members), and kiwifruit (18 members) and slightly lower than that in apple (26 members). The genome sizes of these plants (grapevine is ∼490 Mb, coffee is ∼710 Mb, apple is ∼742 Mb, and kiwifruit is ∼616 Mb) are significantly smaller than the tea plant genome [40,41,42,43]. The phenomenon indicated that the number of *MPK* genes is irrelevant to the size of the plant genome.

The sequence alignment and phylogenetic tree analysis revealed four distinct groups, which were consistent with the MPKs previously identified in *Arabidopsis* [3], rice [14], poplar [38], and sunflower [17]. In group A, tea plants had four extra copies of MPK3 that might have been because of gene duplications. Such extra gene copies have also been observed in sunflowers [17] and apples [44]. Four copies of CsMPK4 were also found in group B. In plants, gene duplications play vital roles in the genomic expansion and diversification of gene function [45]. Two rounds of whole genome duplication events have occurred in the tea tree genome, which resulted in the expansion of several gene classes [22,23]. More copies of CsMPKs might suggest that their capabilities were broken down into more detailed functions.

The exon/intron structure plays an important role in organismal evolution [46]. In the study, we observed that the positions, sizes, and sequences of the introns differed between *CsMPK* genes (Figure 3). Members in group C had the fewest introns, while group D possessed the most introns. Similar exon/intron structures were found in apples [44]. Generally, genes with fewer introns could be rapidly activated in response to environmental stresses [47]. MPK genes are one of the rapidly induced genes under different environmental challenges [3]. The less of introns fit in with the needs for the rapid activation of *MPK* genes. In this study, we found that group A members with fewer introns were induced by various stress treatments, whereas genes with many introns, such as *CsMPK4-4*, *CsMPK9-1*, and *CsMPK16-1*, did not show any significant expression changes under cold, heat, and drought stresses (Figure 5). In addition, CsMPK members with similar conserved motifs and exon/intron structure showed a tendency to cluster into one subgroup (Figures 2 and 3), which implied functional consistency among these CsMPKs in the same subfamily. This correlation between motif arrangement and intron numbers supported the previous classification of the *CsMPK* genes. These results might provide an excellent reference to explore the functions of the CsMPKs.

Numerous reports demonstrated that plant *MPK* genes play important roles in determining response to abiotic stresses [48]. For example, *AtMAPK3*, *AtMAPK4*, *ZmMAPK1*, and *SiMAPK7* were reported to regulate abiotic stress responses [49,50,51]. In this study, the expression levels of the *CsMPK3-1*, *CsMPK3-3*, and *CsMPK3-4*, which were the orthologue of the *AtMAPK3* gene, were induced by cold (Figure 5). Furthermore, these genes possessed at least four types of *cis*-elements in the promoter region (Figure 4), suggesting that they were related to the transcriptional control of stress responses. Notably, *CsMPK3-2* was significantly upregulated under cold and heat combined with drought, implying that *CsMPK3-2* might be an important regulator in response to abiotic stresses. The expressions of *CsMPK3-1*, *CsMPK3-2*, *CsMPK3-3*, and *CsMPK3-4* were downregulated under heat stress. These results are similar to the previous report in which the *SlMPK3* was proposed to negatively regulate heat stress in tomato plants [52]. However, *CsMPK3-5* had nine types of *cis*-elements and fewer introns, and no change was found in the expression level. The results suggested that the *cis*-elements of gene were not all of decisive factor in responding to stresses, or they could be induced by other stresses. *CsMPK4-3*, a member of group B, was also induced by drought stress, consisting of the *AtMPK4* [53]. The *CsMPK* genes belonging to group D have not been as well studied as those of groups A and B. In the present study, the expression of *CsMPK19-1* and *CsMPK19-2* was increased in cold and heat combined with drought stress, and *CsMPK15* and *CsMPK9-2* were upregulated under drought stress, suggesting the possible roles of these *CsMPK* genes in abiotic stress responses. In addition, we found that most *CsMPK* genes possessed at least one hormone-responsive *cis*-element, including ABA, JA, and ethylene-responsive elements (Table S3). The relationship between MAPK signaling pathways and ABA, JA, SA, and ethylene in plant abiotic stress responses was characterized [54,55]. However, little is known about how the *CsMPK* genes respond to phytohormones in tea plant. These questions need further research. The regulatory mechanism of *CsMPK* genes under abiotic stress is complex. More research is needed to explore the specific functions of the CsMPK family genes in tea plants through additional experiments.

## 5 Conclusions

In this study, a genome-wide analysis of *CsMPK* family genes in tea plant was performed, and 21 putative *CsMPK* genes were identified. The identified *CsMPK* genes were classified into four subfamilies (groups A, B, C, and D). The gene structure and conserved motifs of the *CsMPK* genes supported the classification results. Numerous *cis*-elements associated with abiotic stresses were found in the promoter regions of *CsMPK* genes. Further expression profile analysis using qRT-PCR showed that most *CsMPK* genes have a positive or negative response to cold, drought, or heat stresses, indicating that *CsMPK* genes play important roles in abiotic stress response. This work provided useful information on the *CsMPK* family genes of tea plant and will contribute to investigating the function and regulatory mechanism of *CsMPK* genes.

## Supplementary Material

Supplementary Figure
