# Supplementary material for: Genome-wide identification of MAPK family genes and their response to abiotic stresses in tea plant (Camellia sinensis)
Source: Open Life Sci. 2022 Sep 8;17(1):1064–74. doi: 10.1515/biol-2022-0466 (PMC9462544; doi:10.1515/biol-2022-0466)
Supplement: Supplementary Figure [file biol-2022-0466-sm.pdf]

Supplementary Materials

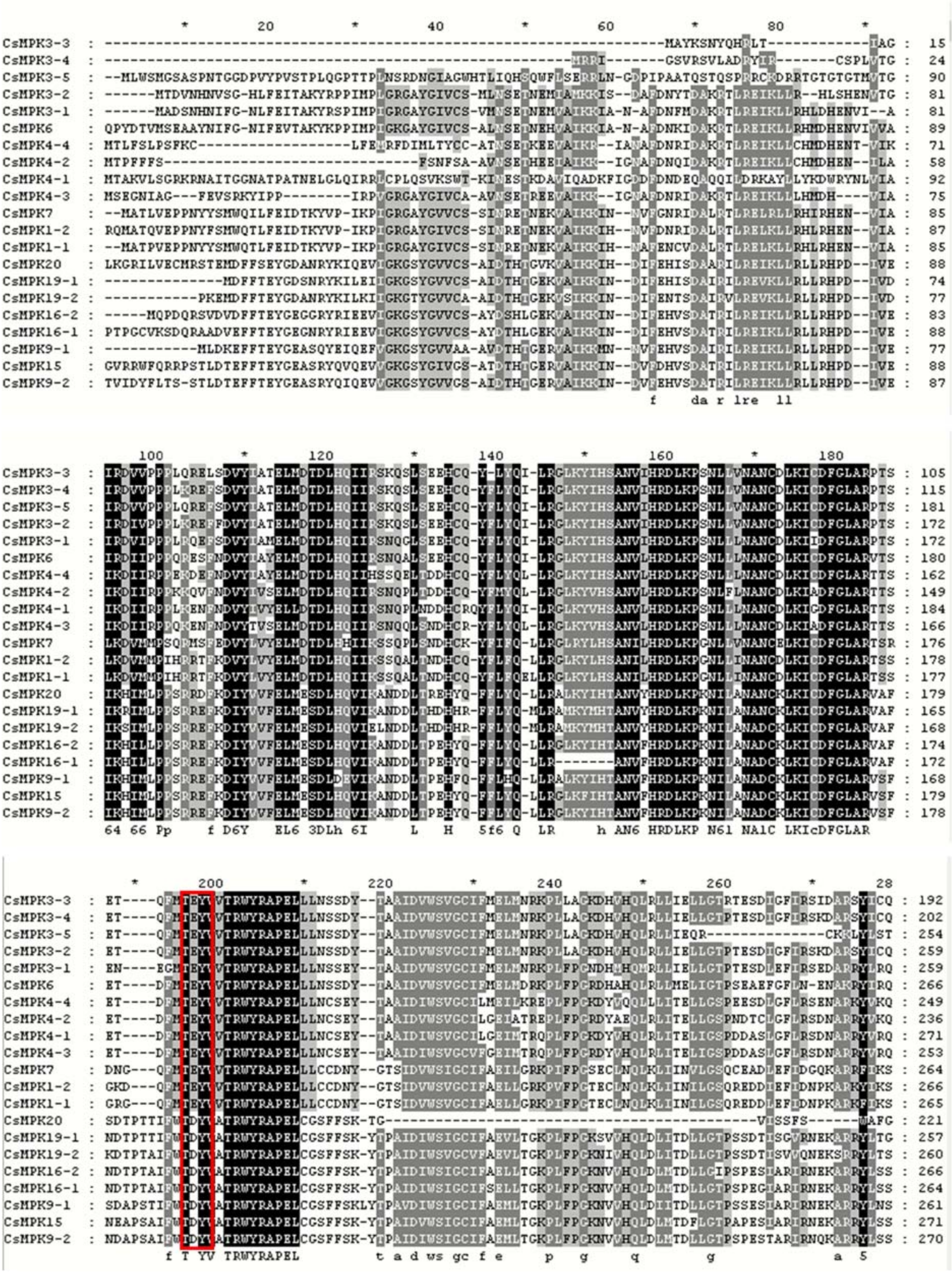

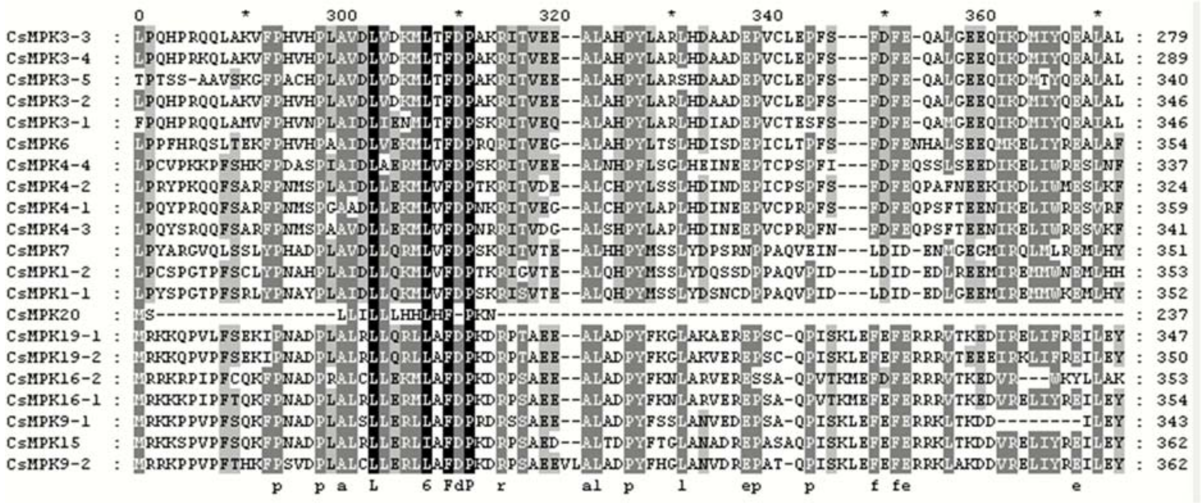

Figure S1: Protein sequence multi-alignment of CsMPKs. Alignment was performed using the ClustalW program. The TXY phosphorylation sites are indicated by red box. Conservative sequences are highlighted by gray or black shading.

Motif 1

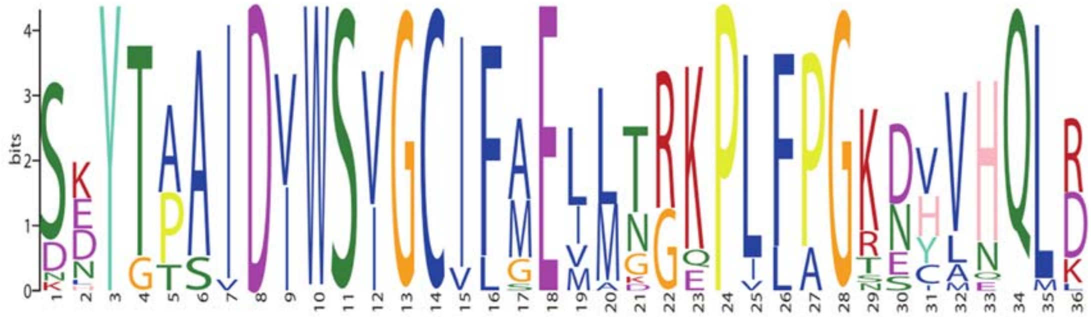

Motif 2

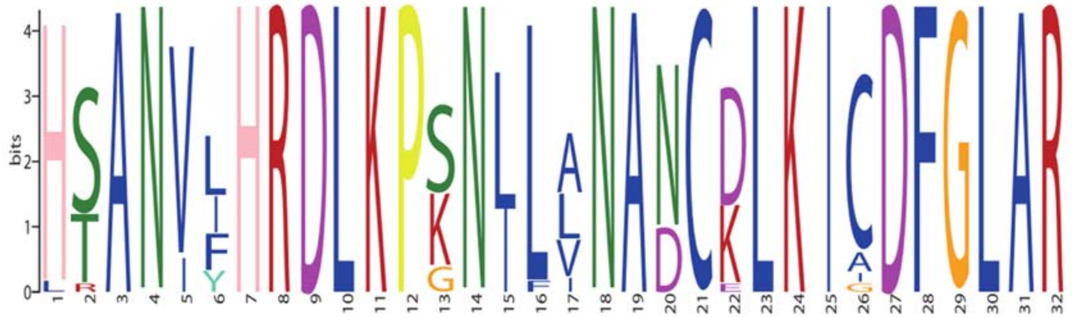

Motif 3

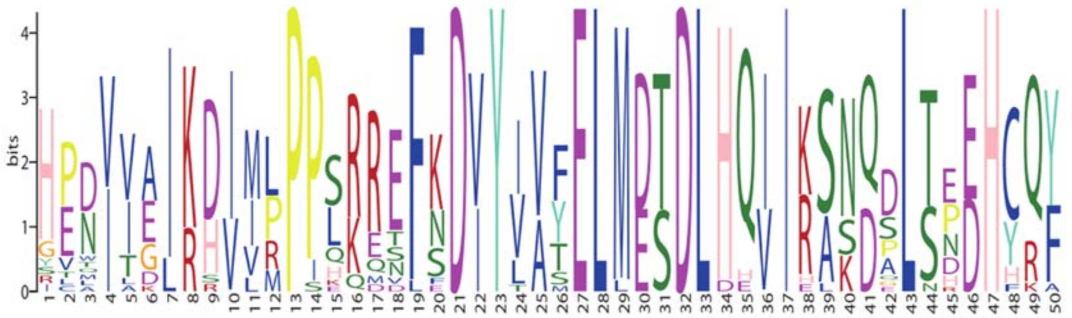

Motif 4

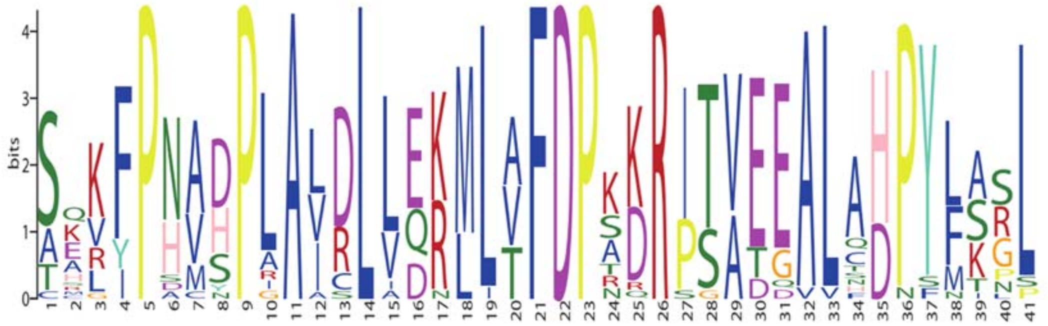

Motif 5

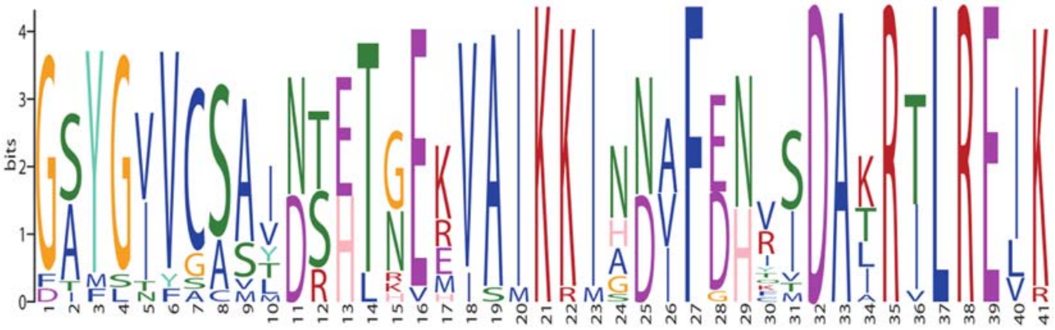

Motif 6

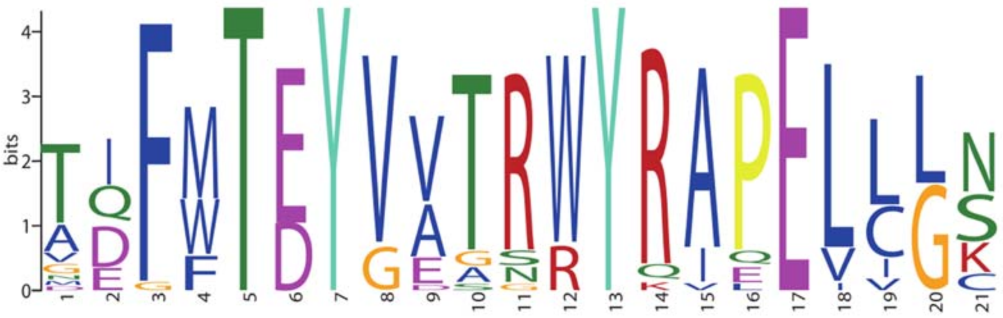

Motif 7

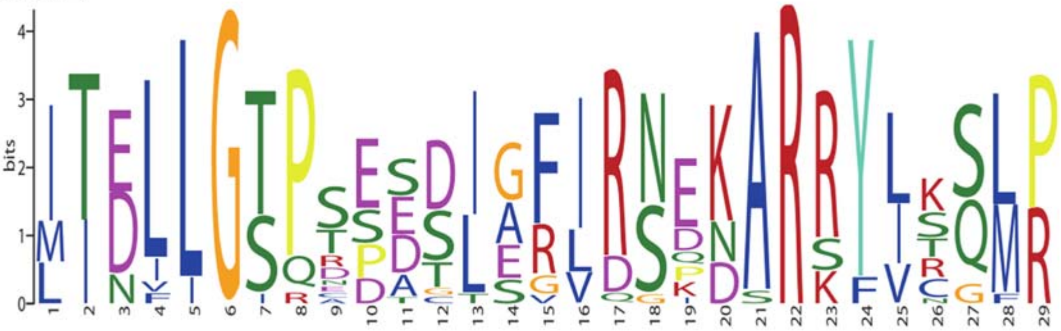

Motif 8

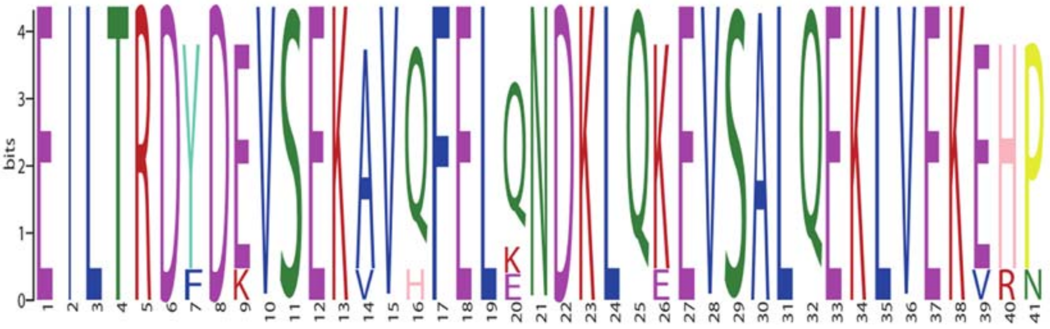

Motif 9

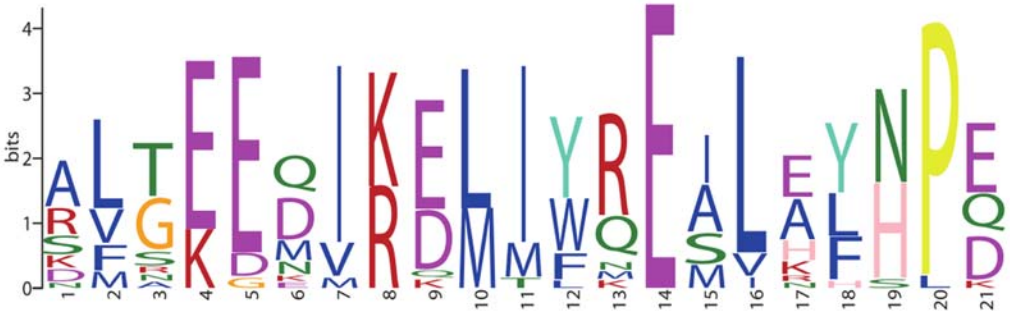

Motif 10

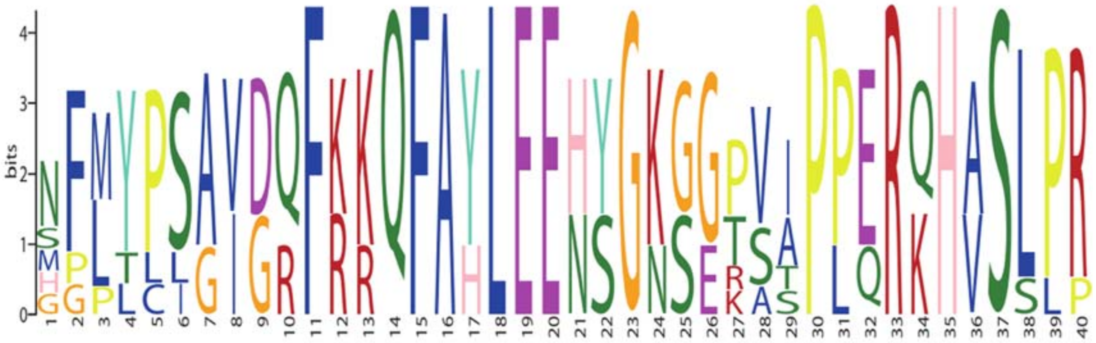

Motif 11

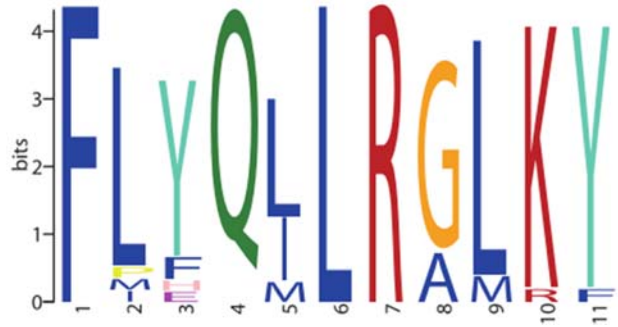

Figure S2: The sequence information of 11 conserved motifs of CsMPKs in tea plant detected by the online tool MEME. (Color figure online).

Table S1: The protein sequences of CsMPKs in tea plant

>CsMPK3-3
MAYKSNYQHRLTIAGIRDVVPPPLQRELSDVYIATELMDTDLHQIIRSKQSLSEEHCQYL
YQILRGLKYIHSANVIHRDLKPSNLLVNANCDLKICDFGLARPTSETQFMTEYVVTRWYR
APELLLNSSDYTAAIDVWSVGCIFMELMNRKPLLAGKDHVHQLRLLIELLGTRTESDIGF
IRSIDARSYICQLPQHPRQQLAKVFPHVHPLAVDLVDKMLTFDPAKRITVEEALAHPYLA
RLHDAADEPVCLEPFSFDFEQALGEEQIKDMIYQEALALNPEYA
>CsMPK3-4
MRRIGSVRSVLADRYIRCSPLVTGIRDVVPPPLKREFSDVYIATELMDTDLHQIIRSKQS
LSEEHCQYFLYQILRGLKYIHSANVIHRDLKPSNLLVNANCDLKICDFGLARPTSETQFM
TEYVVTRWYRAPELLLNSSDYTAAIDVWSVGCIFMELMNRKPLLAGKDHVHQLRLLIELL
GTPTESDIGFIRSKDARSYICQLPQHPRKQLAKVFPHVHPLAVDLVDKMLTFDPAKRITV
EEALAHPYLARLHDAADEPVCLEPFSFDFEQALGEEQIKDMIYQEALALNPDSVTLASQG
WVNNLIVFLIGQFNVKSINATQIFNVINGCVTMFPILGAIVADSFLGSFFVIWISSLISV
LGIILLILSATLDSLRPPPCGNGSNLCITPSKLQFAVLYLGITLATIGAAGTRFTLGAMG
ADQFDNPKHQGSYFNWFIFTLYMASIISSTAIIYVEDSVSFGLGFGLCAAANVIGLVIFV
LGKRFYRHVKPQGSPFTSLARVFVAAIRKRKILLSQRSEDYYNKIHDGVTMMAVTTPTKT
FNFLNRAALKTEGDIRADGSIAKLWNLCTVQQVEDLKALIKIAPLWSSGIFLSTPLAIQY
SLATLQALTMDRHLGPHFKIPPGSMAVFIMIATCITIVLLDRFLYAAWQKLTSRFPTPLQ
RVGVGHVLCVLSMALSAIVESKRLKIARSHNLQDQSVLIVPMSAFWLVPQLALAGIGEAF
HFPGNTALFYQEFPMSLKSTSTAMVAMFVGIAYYVSTALVSGVQRTTGWLPNNINHGRMD
NMYWLLFVIGVVNFGYFVVCAWLYKYQGVAKVVDENPSSDDK
>CsMPK3-5
MLWSMGSASTGPGTGTGMVLVRTAIERRPNTGGDPVYPVSTPLQGPTTPLNSRDNGIAGW
SALFVGKHTLIQHSQWFLSERRLNGDPIPAATQSTQSPRRCKDRRTGTGTGTMVTGIRDV
VPPPLQREFSDVYIATELMDTDLHQIIRSKQSLSEEHCQYFLYQILRGLKYIHSANVIHR
DLKPSNLLVNANCDLKICDFGLARPTSETQFMTEYVVTRWYRAPELLLNSSDYTAAIDVW
SVGCIFMELMNRKPLLAGKDHVHQLRLLIEQRCKKLYLSTTPTSSAAVSKGFPACHPLAV
DLVDKMLTFDPAKRITVEEALAHPYLARSHDAADEPVCLEPFSFDFEQALGEEQIKDMTY
QEALALNLEYA
>CsMPK3-2
MTDVNSAGGELVHGGQFIRHNVSGHLFEITAKYRPPIMPLGRGAYGIVCSMLNSETNEMI
AMKKISDAFDNYTDAKRTLREIKLLRHLSHENVTGIRDIVPPPLKREFFDVYIATELMDT
DLHQIIRSKQSLSEEHCQYFLYQILRGLKYIHSANVIHRDLKPSNLLVNANCDLKICDFG
LARPTSETQFMTEYVVTRWYRAPELLLNSSDYTAAIDVWSVGCIFMELMNRKPLLAGKDH
VHQLRLLIELLGTPTESDIGFIRSKDARSYICQLPQHPRQQLAKVFPHVHPLAVDLVDKM
LTFDPAKRITVEEALAHPYLARLHDAADEPVCLEPFSFDFEQALGEEQIKDMIYQEALAL
NPEYA
>CsMPK3-1
MADSNTVAGQFPEFPAIPTHGGQFIQHNIFGNLFEITAKYRSPIMPIGRGAYGIVCSVMN
SETNEMVAIKKIANAFDNFMDAKRTLREIKLLRHLDHENVIAIRDVIPPPLRQEFSDVYI
AMELMDTDLHQIIRSNQGLSEEHCQYFLYQILRGLKYIHSANVIHRDLKPSNLLLNANCD
LKIIDFGLARPTSENEGMTEYVVTRWYRAPELLLNSSEYTAAIDVWSVGCIFMELMNRKP

(*Continued*)

Table S1: *Continued*

LFPGNDHMHQMRLLIELLGTPTESDLEFIRSEDARRYLRQFPQHPRQQLAMVFPHVNPLA
IDLIENMLTFDPSKRITVEQALAHPYLARLHDIADEPVCTESFSFDFEQAMGEEQIKDMI
YQEAIALNPEFA
>CsMPK6
MDASAQPYDTVMSEAAPPPPQSDPPPQPPPAAGIEHIPATLSHGGRFIQYNIFGNIFEVT
AKYKPPIMPIGKGAYGIVCSALNSETNEHVAIKKIANAFDNKIDAKRTLREIKLLRHMDH
ENVINFGSSFPKMTLNVNSFTQCCERRSEVQAVRLKGPKARLQPHSITTYIIQPNLNQST
QIPNLSKKSVSSLPLGSSLSISTSISIEVSSPPPPPPPPLLLCWSLVAGCWVVAIRDIIP
PPQRESFNDVYIAYELMDTDLHQIIRSNQALSEEHCQYFLYQILRGLKYIHSANVLHRDL
KPSNLLLNANCDLKICDFGLARVTSETDFMTEYVVTRWYRAPELLLNSSDYTAAIDVWSV
GCIFMELMDRKPLFPGRDHAHQLRLLMELIGTPSEAEFGFLNENAKRYIRQLPPFHRQSL
TEKFPHVHPAAIDLVEKMLTFDPRQRITVEGALAHPYLTSLHDISDEPICLTPFSFDFEN
HALSEEQMKELIYREALAFNPEYQQIM
>CsMPK4-4
MTLFSLPSFKCLFEMRFDIMLTYCCATNSETKEEVAIKRIANAFDNRIDAKRTLREIKLL
CHMDHENTTIVQVIKIKDIIRPPEKDEFNDVYIAYELMDTDLHQIIHSSQELTDDHCQYF
LYQLLRGLKYIHSANVLHRDLKPSNLLLNANCDLKICDFGLARTTSETDFMTEYVVTRWY
RAPELLLNCSEYTAAIDIWSVGCILMEILKREPLFPGKDYVQQLLLITELLGSPEESDLG
FLRSENARKYVKQLPCVPKKPFSHKFPDASPIAIDLAERMLVFDPSKRITVEEALNHPFL
SGLHEINEEPTCPSPFIFDFEQSSLSEEDIKELIWRESLNFSPDEFFNVLTGCWEAVVVG
IILGLCFILSSRVCPMMNLYAKKKKSDLKIQGAKKKAMRFSQIFVKASSSSSHSRNRSAS
ASSLLLSTASRSFSHLRSPSQRPSFGIAFDIDGVILRGNTPIGGSPQALGRLYHDSFGTL
KVPYVFLTNGEPFVSSCYTRPYTFQTAGKERSYFMGTKEVLLVDVAYVVRFENDLIIAVG
KGEPATVMSEYGFKNAFSIDEYASCFDNIDPLAQFKKWTTGHVNQNNTIRSTACTQRVHA
AFIVSDSVDWSRDIQVLCDILRTGGFPGREIAHQPSLYFANDDLEYQAVFPSERLGMGAF
RIALESIFNSIHPNALEYTSFGKPNPSVFRNAETVLMETVPSSYQSANHVTGGNHVFRTL
YMIGDNPSVDIKGAKQAGHPWFSILTRTGVFKGKKNHAEHPADLVGELTLYICSNFSFKF
HFSLETIILLLSTFASQT
>CsMPK4-2
MTPFFFSFSNFSAAVNSETHEEIAIKKIGNAFDNQIDAKRTLREIKLLCHMDHENILAIK
DIIRPPKKQVFNDVYIVSELMDTDLHQIIRSNQPLTDDHCQYFMYQLLRGLKYVHSANVL
HRDLKPSNLFLNANCDLKIADFGLARTTSETDFMTEYVVTRWYRAPELLLNCSEYTAAID
VWSVGCILGEIATREPLFPGRDYAEQLRLITELLGSPNDTCLGFLRSDNARRYVKQLPRY
PKQQFSARFPNMSPLAIDLLEKMLVFDPTKRITVDEALCHPYLSSLHDINDEPICPSPFS
FDFEQPAFNEEKIKDLIWMESLKFNPDPTH
>CsMPK4-1
MMLHIRRQMKMEMKVVEGGKSAEEDDEDDVAKSVEGSINGNHTVEGLSDDVDGGQSLERQ
QQPYPYLADDALGELKVAYEALKEHNISSEAANIEVIILYEFIKQHVCDIEAKKGELLGL
YEAVRQHSTILGAENSELVKKLGDYQPRISVLDRQLDEMHSSSDEMASSISNQVEVLHKE
VAERASILEQEWNSTVAQVVQTVEKLDALIRSLFSTASVDAATKVIDNLQEKLEASGKDH
TAMCSSYKDMIEKFNDLHRKNELAIGVLHGVHGNLQRLVNDSCQHVEQSQIINQNENLID

(*Continued*)

Table S1: *Continued*

PLHLDNYDTLMEQLAVLLGERLQFKSMNDKLNLELIDMAKEMEKLKKTGLDSDTILKLVE
DVEGAVKLNGTGIDSEKLESRLQRLISFLSDSLEILTRDYDEVSEKAVQFELQNDKLQKE
VSALQEKLVEKEHPSDSLEILTRDYDEVSEKAVHFELKNDKLQKEVSALQEKLVEKEHPS
DSLEILTRDYDEVSEKAVQFELQNDKLQKEVSALQEKLVEKEHPSDSLEILTRDYDEVSE
KAVQFELQNDKLQKEVSALQEKLVEKEHPSDSLEILTRDYDEVSEKAVQFELQNDKLQKE
VSALQEKLVEKEHPSDSLEILTRDYDEVSEKAVQFELQNDKLQKEVSALQEKLVEKEHPS
DSLEILTRDYDEVSEKAVQFELQNDKLQKEVSALQEKLVEKEHPSDSLEILTRDFDKVSE
KVVQFELENDKLQEEVSALQEKLVEKVRNQEHIEGEIRRLEDLVHDLLKDSVTEDVVSAG
SSTKCLEQLLKKLSEKYTALALEKPILVDSADEKNTKITRPRPMTAKVLSGRKRSTRVRG
RTLGKGVQKRIARKKGEKLHVYVNRVLNAITGGNATPATNELGLQIRRLCPLQSVKSWTK
INESTKDAVIQADKFVIGDDFDNDEQAQQILDRKAYLLYKDWRYNLKQEFLELEEKGVDD
PYSHPPSGVSLDDWRYLIDVAWKDESHLKKEEGCTFKPEWAVAYEALKEHNISSEAANIE
VIILYEFIKQHVCDIEAKKGELLGLYEAVRQHSTILGAENSELVKKLGDYQPRISVLDRQ
LDEMHSSSDEMASSISNQVEVLHKEVAERASILEQEWNSTVGQVVQTVEKLDALIRSFFL
AWTYVAVIAIKDIIRPPLKENFNDVYIVYELLDTDLHQIIRSNQPLNDDHCRALYTASGN
ELFVKGYVSFFIHQGDVRNLTSNMQSVCVNMTSVRADCIVHLIDSSILLMQYFLYQILRG
LKYVHSANVLHRDLKPSNLLLNANCDLKIGDFGLARTTSETDFMTEYVVTRWYRAPELLL
NCSEYTAAIDIWSVGCILGEIMTRQPLFPGKDYVHQLRLITELIGSPDDASLGFLRSDNA
RRYVRQLPQYPRQQFSARFPNMSPGAADLLEKMLVFDPNKRITVEGALCHPYLAPLHDIN
EEPVCPRPFSFDFEQPSFTEENIKELIWRESVRFNPDPPISH
>CsMPK4-3
MSEGNIAGIPTHGGRYVQYNVYGTLFEVSRKYIPPIRPVGRGAYGIVCAAVNSETREEVA
IKKIGNAFDNRIDAKRTLREIKLLLHMDHENVIAIKDIIRPPQKENFNDVYTVSELMDTD
LHQIIRSNQQLSNDHCRYFLYQLLRGLKYVHSANVLHRDLKPSNLLLNANCDLKIADFGL
ARTTSETDFMTEYVVTRWYRAPELLLNCSEYTAAIDIWSVGCVFGEIMTRQPLFPGRDYV
HQLRLITELIGSPDDASLGFLRSDNARRYVRQLPQYSRQQFSARFPNMSPAAVDLLEKML
VFDPNRRITVDGALSHPYLAPLHDINEEPVCPRPFNFDFEQPSFTEENIKELIWRESVKF
NPDPPIH
>CsMPK7
MATLVEPPNGVKPLGKHYYSMWQILFEIDTKYVPIKPIGRGAYGVVCSSINRETNEKVAI
KKINNVFGNRIDALRTLRELRLLRHIRHENVIALKDVMMPSQRMSFEDVYLVYELMDTDL
HHIIKSSQPLSNDHCKYFIFQLLRGLRYLHSANILHRDLKPGNLLVNANCELKICDFGLA
RTSRDNGQFMTEYVVTRWYRAPELLLCCDNYGTSIDVWSIGCIFAEILGRKPIFPGSECL
NQLKLIINVLGSQCEADLEFIDGQKARRFIKSLPYARGVQLSSLYPHADPLAVDLLQRML
VFDPSKRITVTEALHHPYMSSLYDPSRNPPAQVEINLDIDENMGEGMIRQLMLREMLHYH
PEAACANT
>CsMPK1-2
MLKDLKNVIARQMATQVEPPNGIKSVGKHYFSMWQTLFEIDTKYVPIKPIGRGAYGIVCS
SINRETNEKVAIKKIHNVFDNRIDALRTLRELKLLRHLRHENVIALKDVMMPIHRRTFKD
VYLVYELMDTDLHQIIKSSQALTNDHCQYFLFQLLRGLKYLHSANILHRDLKPGNLLINA
NCDLKICDFGLARTSSGKDQFMTEYVVTRWYRAPELLLCCDNYGTSIDVWSVGCIFAELL

(*Continued*)

Table S1: *Continued*

GRKPVFPGTECLNQLKLIINILGSQREDDIEFIDNPKARKYIKSLPCSPGTPFSCLYPNA
HPLAIDLLQKMLVFDPTKRIGVTEALQHPYMSSLYDQSSDPPAQVPIDLDIDEDLREEMI
REMMWNEMLHHHPEAAPANMEVNQ
>CsMPK1-1
MATPVEPPNGINSPGKHYYSMWQTLFEIDTKYVPIKPIGRGAYGIVCSSINRETNEKVAI
KKIHNAFENCVDALRTLRELKLLRHLRHENVIALKDVMMPIHRRTFKDVYLVYELMDTDL
HQIIKSSQALTNDHCQYFLFQEDSVVLKLVMKDSVNGQTFFGLDLCRDSGALGQNMEVTT
IGKSNGNFPEQLLRGLKYLHSANILHRDLKPGNLLINANCDLKICDFGLARTSSGRGQFM
TEYVVTRWYRAPELLLCCDNYGTSIDVWSVGCIFAELLGRKPIFPGTECLNQLKLIINIL
GSQREDDLEFIDNPKARKFIKSLPYSPGTPFSRLYPNAYPLAIDLLQKMLVFDPSKRISV
TEALQHPYMSSLYDSNCDPPAQVPIDLDIDEDLGEEMIREMMWKEMLHYHPEAATANVDV
S
>CsMPK20
MIVGGGEVLKGRILVECMRDLIRFPCFVKNSKRVRWDCSFVIRSRVLNKQTMQQDHGKKN
STEMDFFSEYGDANRYKIQEVIGKGSYGVVCSAIDTHTGVKVAIKKIHDIFEHISDAARI
LREIKLLRLLRHPDIVEIKHIMLPPSRRDFKDIYVVFELMESDLHQVIKANDDLTREHYQ
FFLYQLLRALKYIHTANVYHRDLKPKNILANANCKLKICDFGLARVAFSDTPTTIFWTDY
VATRWYRAPELCGSFFSKTGVISSFSWAFGMSLLILLLHHLHFPKN
>CsMPK19-1
MDFFTEYGDSNRYKILEIIGKGSYGVVCSAIDTHTGEKVAIKKINDIFEHISDAIRILRE
VKLLRLLRHPDIVDIKRIMLPPSRREFKDIYVVFELMESDLHQVIKANDDLTHDHHRFFL
YQMLRAMKYMHTANVYHRDLKPKNILANANCKLKICDFGLARVAFNDTPTTIFWTDYVAT
RWYRAPELCGSFFSKYTPAIDIWSIGCIFAEVLTGKPLFPGKSVVHQLDLITDLLGTPSS
DTISGVRNEKARRYLTGMRKKQPVLFSEKIPNADPLALRLLQRLLAFDPKDRPTAEEALA
DPYFKGLAKAEREPSCQPISKLEFEFERRRVTKEDIRELIFREILEYHPQLLKDYLAGNE
GPNFLYPSAIGQFRKQFAYLEENSGKSGPVIPPERKHVSLPRSTVNSSTVPPRTNQNLAS
CGSRQVTEESCNSIRVTDAISGGNFVKPSRPPPKVPAAKPGRVVGPVLPYENGRNINDVY
DARAFLRNGVVPQGISPQYAARTNTVNHAKSSTEIDRNSAQARQLPAQCNGVTKQSPQIA
IDINANTYYQPQVKSSQLNDQIAINAKLLQEQSQLGAIGAAAVAVAARREVGAVQLGLS
>CsMPK19-2
MQRDQRKKSPKEMDFFTEYGDANRYKILKIIGKGTYGVVCAAIDTHTGEKVSIKKINDIF
ENTSDAIRVLREVKLLRLLRHPDIVDIKSIMLPPSRREFKDIYVVFELMESDLHQVIELN
DDLTHDHHRFFLYQMLRAMKYMHTANVYHRDLKPKNILANADCKLKICDFGLARVAFKDT
PTAIFWTDYVATRWYRAPELCGSFFSKYTPAIDIWSVGCVFAEVLTGKPLFPGKNIVHQL
DLITDLLGTPSSDTISVVQNEKSRRYLTSMRKKQPVPFSEKIPNADPLALRLLQRLLAFD
PKDRPTAEEALADPYFKGLAKVEREPSCQPISKLEFEFERRRVTEEEIRKLIFREILEYH
PQLLKYYLAGNEGANFLYPSAVGQFRKQFAYLEENSGKSGPVIPPERKHVSLPRAVGQFR
KQFAYLEENSGKSGPIIPPERKHYTPAIDIWSIGCIFAEVLTGKPLFPGKSVVHQLDLIT
DLLGTPSSDTISGVRNEKARRYLTGMRKKQPVLFSEKIPNADPLALRLLQRLLAFDPKDR
PTAEEALADPYFKGLAKAEREPSCQPISKLEFEFERRRVTKEDIRELIFREILEYHPQLL
KDYLAGNEGPNFLYPSAIGQFRKQFAYLEENSGKSGPVIPPERKHVSLPRSTVNSSTVPP

(*Continued*)

Table S1: *Continued*

RTNQNLASCGSRQVTEESCNSIRVTDAISGGNFVKPSRPPPKVPAGMVDLVKTHELKGLI
LKETELGPEKRKQVGRLVESIGFILGWINTVLLCRPLRNALTVSNGDLKRHEVPARADPV
VRVVSNPNSFSP
>CsMPK16-2
MQPDQRKKVYVSSSMPMFSSNLGPSKGYVLHGSVDVDFFTEYGEGGRYRIEEVIGKGSYG
VVCSAYDSHLGEKVAIKKINDIFEHVSDATRILREIKLLRLLRHPDIVEIKHILLPPSRR
EFKDIYVVFELMESDLHQVIKANDDLTPEHYQFFLYQLLRGLKYIHTANVFHRDLKPKNI
LANADCKLKICDFGLARVAFNDTPTAIFWTDYVATRWYRAPELCGSFFSKYTPAIDIWSI
GCIFAELLTGKPLFPGKNVVHQLDLMTDLLGIPSPESIARIRNEKARRYLSSMRRKRPIP
FCQKFPNADPRALCLLEKMLAFDPKDRPSAEEALADPYFKNLARVERESSAQPVTKMEFD
FERRRVTKEDVRWKYLLAKCLAVAFLLVFQEDSMGMTCIAVDQFKKQFAYLEEHYGNGGT
VAPPERQHASLPRPCVLYSSDNSMQSSAEVTNDLTKCCIKEVEKPHVDRISGIPKTRLPL
QVPPTVGVNFFYDHRFSGAAAARPGKVVGSVLRYNNCGAEALEHRRMVRNPAVPTQYAVS
TSRRNPGSKNERREDGVEGSSVLQPKPPQYVSRKVAAAQGGSGSHWH
>CsMPK16-1
MGLLRTFLLPTPGCVKSDQRASISDLVWISDDSIRVFRKHGCHRRLMSDSARTLLGFAVR
NLSLKMEIASPTERENRRRRRGNEIIIAKNPNYKELETQFKSTSRNPNLEQKWKVVWIER
KGIGMSEVAADVEFFTEYGEGNRYRIEEVIGKGSYGVVCSAYDTHLGEKVAIKKINDIFE
HVSDATRILREIKLLRLLRHPDIVEIKHILLPPSRREFKDIYVVFELMESDLHQVIKAND
DLTPEHYQFFLYQLLRANVFHRDLKPKNILANADCKLKICDFGLARVAFNDTPTAIFWTD
YVATRWYRAPELCGSFFSKYTPAIDIWSIGCIFSELLTGKPLFPGKNVVHQLDLMTDLLG
TPSPEGIARIRNEKARRYLSSMRKKKPIPFTQKFPNADPLALRLLERMLAFDPKDRPSAE
EALADPYFKNLARVEREPSAQPVTKMEFEFERRRVTKEDVRELIYREILEYHPKMLKEYL
EGEEPTGFMYPSAVDQFKKQFAYLEEHYGNGGTAAPPERQHASSLPRACVLYSDNSAQNS
TEVTNDLSKCCIKEVERPHMDRTSGIPMTRMPPQVPPSIQAGGAARPGKVVSSVLRYNNC
GAAAAAAETVEQRRMVRNPVVPTQYAVSSSSYARRNSACKNERGGEDGVEGSNGLQPKPQ
YMPRKVAAAQGGSGSQWLCTSLTRSDLNLRICPQKFTNPLSVFHSKPQFLNRNCFKSPFQ
SSISATHRLISSIYRMGDSRRPPTVPIPDVEIADKSELYRALEASLGSSFSSNPLAPNPN
PLIIVISGPSGVGKDAVIKGLREVREGIHFVVTATSRAKRPGEVEGKDYFFVSKEEFLSM
VERDELLEYALVYGDYKGIPKQQIREFMAKGHDIVLRVDIQGAATLRRILGNSAVFIFLV
AESESALVKRLIGRKTETKETLLVRVATAREEVKHLRNFDYVVVNAEGKLESAVKLVESI
IDAEKAKVWQRTAVI
>CsMPK9-1
MLDKEFFTEYGEASQYEIQEFVGKGSYGVVAAAVDTHTGERVAIKKMNNVFEHVSDAIRI
LREIKLLRLLRHPDIVEIKHIMLPPSRREFKDIYVVFELMESDLDEVIKANDDLTPEHFQ
FFLHQLLRALKYIHTANVFHRDLKPKNILANADCKLKICDFGLARVSFSDAPSTIFWTDY
VATRWYRAPELCGSFFSKVTQSGFNNLSHCIKVCSNPVSCSGWLSEPHSEQPIQLEHVLN
LYTPAVDIWSIGCIFAEMLTGKPLFPGKNVVHQLDIITDLLGTPSSESIARIRNEKARRY
LNSMRKKPPVPFSQKFPNADPLALSLLERLLAFDPRDRSSAEEALADPYFSSLANVEDEP
SAQPISKLEFEFERRKLTKDDILEYHPQMLQEYLDGVDRTSFMYPRSVLCGLRFSFDPRI
YAVFEKVNPQPPAVRFAPTSLDLRIRGKKVILTGFPNHSKKGIRLLMQLILPLLMLLSQR
ILPMECNREPEIRCEQSVEGAELRAFTFYYISKAHSL

(*Continued*)

Table S1: *Continued*

>CsMPK15
MGSGTFVDGVRRWFQRRPSSPQPNNTQNLNNSQPLVSGFQGEDHLNVVEDFDFSGLKIIK
VPKRINFHPTSMDPQKKTTLDTEFFTEYGEASRYQVQEVVGKGSYGIVGSATDTHTGERV
AIKKINDVFDHVSDATRILREIKLLRLLRHPDIVEIKHIMLPPSRREFKDIYVVFELMES
DLHQVIKANDDLTPEHYQFFLYQLLRGLKFIHTANVFHRDLKPKNILANADCKLKICDFG
LARVSFNEAPSAIFWTDYVATRWYRAPELCGSFFSKYTPAIDVWSIGCIFAEMLTGKPLF
PGKNVVHQLDLMTDFLGTPAPESIARIRNEKARRYLSSMRKKSPVPFSQKFPNADPLALR
LLERLIAFDPKDRPSAEDALTDPYFTGLANADREPASAQPISKLEFEFERRKLTKDDVRE
LIYREILEYHPQMLQEYLRGGDQTSFMYPSGIDRFKRQFAHLEEHYGKGEKSTPLQRQHA
SLPRERVCGQKDETISQNDDLEKRTAASVATTIQSSPKESEESENANTNAQSGLNKPNYS
ARTLLKSASISGSKCVVVQAKKDSKEEPIAEHEEVDELTQKLAAINS
>CsMPK9-2
MTQTVIDYFLTSSAPCPGLDCLSYPSTGYTLDTEFFTEYGEASRYQIQEVVGKGSYGVVG
SAIDTHTGERVAIKKINDVFEHVSDATRILREIKLLRLLRHPDIVEIKHIMLPPSRREFK
DIYVVFELMESDLHQVIKANDDLTPEHYQFFLYQLLRGLKYIHTANVFHRDLKPKNILAN
ADCKLKICDFGLARVSFNDAPSAIFWTDYVATRWYRAPELCGSFFSKYTPAIDIWSIGCI
FAEMLTGKPLFPGKNVVHQLDLMTDLLGTPSPESTARIRNQKARRYLSSMRRKPPVPFTH
KFPSVDPLALCLLERLLAFDPKDRPSAEEVVFHNSNILPLALADPYFHGLANVDREPATQ
PISKLEFEFERRKLAKDDVRELIYREILEYHPQMHQEYLCGGDQTSFMYPRPDLPPLLCA
SAILAFVLAVHPPLLLGVDRFKRQFAHLEEHYGKGERSSPLQRQHASLPRERVCVPKDEA
TSQNNDFEVRTTASVATTLKSPPSENANTDAQNGPSKPNYSARSLLKSASISASKCIGAK
GRKDSEGEEIAEQNEEVDASSQKVAALCA

Table S2: The primer sequences of the CsMPK genes in tea plant for quantitative RT-PCR

Name	Forward primers (5′to 3′)	Reverse primers (5′to 3′)
CsMPK4-2	TTCCAATTTCAGTGCTGCTG	TGAAGATCGGTGTCCATCAA
CsMPK3-1	ACCCACTGAATCGGATCTTG	GACCGGTTCGTCAGCTATGT
CsMPK19-1	CGAAATGAGAAGGCAAGGAG	GTCGCCTCTCGAACTCAAAC
CsMPK4-1	TTCCACAATACCCACGACAA	TCAGTGAATGATGGCTGCTC
CsMPK9-1	CCACAGATGCTGCAGGAGTA	GTTCGGAAACCCAGTGAGAA
CsMPK6	CTTCAGCTCAGCCCTACGAC	GAATTCAAAGCCGAGCAGAC
CsMPK15	ACAGTTTGCCCATCTTGAGG	TGATTCCTCGGATTCTTTGG
CsMPK19-2	GCAGGAATGGTTGATCTGGT	GAGGCCGACATAAAAGGACA
CsMPK16-2	GTGGAACTGTTGCTCCACCT	AGCACCTGAGAAGCGATGAT
CsMPK7	ATCTCAACCGCTTTCCAATG	TAGCCCGAAGTCGCATATCT
CsMPK3-3	CTTGCGGGTAAAGACCATGT	GATGGACATGTGGGAAAACC
CsMPK3-2	CTTGCGGGTAAAGACCATGT	GATGGACATGTGGGAAAACC
CsMPK20	GTGGAGTGCATGAGGGATCT	TCAATGGCTGAGCAAACAAC
CsMPK4-3	TATACGCCCTCCACAAAAGG	CAATTTGCATTGAGGAGCAA
CsMPK1-2	CCTGGGAACCTTCTCATCAA	AAAGATGCATCCGACAGACC
CsMPK9-2	ATCAGCTTCTTCGTGGCCTA	AGCACGATACCATCGGGTAG
CsMPK4-4	GAGTCTGCCCCATGATGAAT	AGCGAAGGTGTGAGAAGGAA
CsMPK16-1	GTCAGCTCAGTGTTGCGGTA	TTGGATCCTTCGACTCCATC
CsMPK1-1	CCAGAGCTCCTCCTCTGTTG	CCTTGCTTTCGGGTTATCAA
CsMPK3-4	CTTGCGGGTAAAGACCATGT	GATGGACATGTGGGAAAACC
CsMPK3-5	CTTGCGGGTAAAGACCATGT	AGCTGCGTCATGTGATCTTG
Actin	GCCATCTTTGATTGGAATGG	GGTGCCACAACCTTGATCTT

Table S3: The number and composition of promoter elements of each CsMPK gene

	ABRE	TGACG-motif	CGTCA-motif	TCA-element	ERE	GARE-motif	P-box	LTR	MBS	MYB	MYC	ARE
CsMPK1-1		3	3					1		1	1	1
CsMPK1-2	1				1		1	1	1	4	2	
CsMPK3-1	2				2					4	2	
CsMPK3-2				1	2			1	1	3	1	2
CsMPK3-3	1				2						2	3
CsMPK3-4	6	1	1		1	1				3	4	2
CsMPK3-5	1	1	1	1				1	1	6	2	1
CsMPK4-1	2	2	2	1		1			1	4	3	1
CsMPK4-2		1	1	1						2	3	6
CsMPK4-3	2	2	2		5			1	1	2	5	2
CsMPK4-4									1	1	2	1
CsMPK6					3		1	1		4	7	1
CsMPK7					3					1	4	
CsMPK9-1				1				1	2	6	3	4
CsMPK9-2	1	1	1							3	2	6
CsMPK15				2	1		1			6	1	4
CsMPK16-1		1	1		2					4	5	1
CsMPK16-2	2	1	1		1					4	3	1
CsMPK19-1				2			1			4	2	3
CsMPK19-2		2	2	1				2	1	1	3	
CsMPK20					4		1			3	2	5
